# Computing with electromagnetic fields rather than binary digits: a route towards artificial general intelligence and conscious AI

**DOI:** 10.3389/fnsys.2025.1599406

**Published:** 2025-06-25

**Authors:** Johnjoe McFadden

**Affiliations:** Faculty of Health and Medical Sciences, University of Surrey, Guildford, United Kingdom

**Keywords:** consciousness, AI, computing (architecture), neurobiology, neuroscience, electromagnetism, consciousness theory, artificial intelligence

## Abstract

McFadden’s conscious electromagnetic information (CEMI) field theory proposes that the human brain functions as a hybrid digital-EM field computer. The digital computations are implemented by the matter-based neuronal-synaptic network analogous to conventional digital computers operating Boolean-like logic gates nonconsciously and in parallel. Yet neuronal electrical firing and synaptic transmission generate the brain’s immaterial but equally physical endogenous electromagnetic (EM) input into the brain’s CEMI field. The CEMI field is proposed to implement analogue information processing through constructive and destructive wave mechanical interference. The output of this field-based processing is uploaded by EM field-sensitive neurons via voltage-gated ion channels to generate conscious actions. According to the theory, non-conscious brain processing occurs solely within the EM field-insensitive digital neuronal network, enabling fast, parallel computations, but cannot form complex, integrated concepts, so it is limited to specialised functions necessary for tasks like motor coordination. In contrast, conscious thought arises from EM field interactions, where integrated information is encoded and processed holistically to deliver general intelligence and creativity as its output. Because the brain’s EM field is singular, conscious processing occurs serially, allowing our mind to hold only one thought at a time. This paper proposes a route towards developing novel hybrid computers that, like the human brain, similarly operate both modes of computation to deliver general intelligent and potentially conscious AI.

## Introduction

Red lips are not so red as the stained stones kissed by the English dead.

The Glorious Dead, Wilfred Owen.

“What’s the best way to fix a bicycle with a rope caught in its spokes?”

Gary Marcus “Deep learning: a critical appraisal” (Marcus, 2018).

Despite the regularity of predictions to the contrary, it remains the case that the only electronic computers known to possess either general intelligence or consciousness are made of flesh and reside inside humans^1^ skulls. This is a remarkable fact. The Nobel Prize-winning physicist Richard Feynman famously insisted that “what I cannot make, I do not understand.” According to Feynman’s criteria, we understand neither general intelligence nor consciousness. In the spirit of Occam’s razor, the simplest explanation, reducing two problems to one, is that general intelligence is the gift of consciousness and will only be achieved by conscious AI. So, what is consciousness, and what does it do?

The quotation that opened this paper from the WW1 war poet Wilfred Owen illustrates the most distinctive aspect of consciousness: it integrates information. The “binding problem” is understanding “*our capacity to integrate information across time, space, attributes, and ideas*” (Treisman, 1999) within a conscious mind (Treisman, 1999; Hardcastle, 1994; Treisman, 1996; Singer, 2001; Jones, 2016). The problem is often posed in understanding how the disparate components of a visual scene—colours, textures, lines, motion, etc.—are processed in distinct brain regions yet brought together into a unified visual-conscious percept. However, binding is a general feature of consciousness in all its modes, including our understanding of language and text such as poetry. Imagine asking a stand-alone computer (with no access to online literary criticism) to interpret Owen’s line quoted above. It would likely conclude that it highlights the difference in redness between human lips and blood. Our conscious minds know that it is about much more, including the tragedy of young men spilling their blood on stones in a foreign field rather than kissing the lips of their lovers. The single line of poetry captures a depth and breadth of integrated meaning that no digital computer can grasp, as their thoughts are never more than zeros or ones. Conventional computers process but do not bind information.

In the second quotation, AI researcher and pioneer of deep learning, Gary Marcus, laments that AI currently lacks general intelligence, as illustrated by the intractability of problems, such as untangling a rope from the wheel of a bicycle that is, nevertheless, grasped and solved by any infant on their first exposure to the task. Tasks that engage general intelligence, like untangling a rope or understanding a poem, engage the conscious mind.

A curious and often overlooked feature of human cognition is that we possess two kinds of minds. The first is the nonconscious^2^ mind that efficiently executes routine motor tasks such as walking or moving our lips to form words without awareness. Our second mind undertakes computational tasks, such as fixing a bicycle with a rope tangled in its wheels, that are consciously solved by *turning the problem over* in our singular conscious mind. The solution may or may not be is then separately implemented to direct motor actions. Note that there is no obvious difference in complexity between these very different tasks. Note that there is no obvious difference between the complexity of information processing performed by the nonconscious or conscious mind. This is evident when we consider that simple stimuli, such as the pain of stubbing your toe, can instantly override complex conscious thoughts, such as contemplating what a poem means or understanding how a bicycle operates. The kind of general intelligence delivered by nonconscious human minds is thereby unlikely to be acquired simply by increasing the complexity of computers that emulate only the nonconscious mind.

A clue to the nature of the difference between conscious and nonconscious mental processing is that our nonconscious mind is a parallel computer whereas our conscious mind is serial. For example, the nonconscious mind can simultaneously operate the contractions of scores of independent muscles needed to cycle a bicycle whilst we whistle a familiar tune, entirely without awareness of their actions. But try performing long division in your head whilst chatting with a friend. We cannot perform parallel computations in our conscious mind; instead, we must switch between tasks. The problem, as succinctly put by the philosopher Bernard Baars, is to understand how “a serial, integrated and very limited stream of consciousness emerges from a nervous system that is mostly unconscious, distributed, parallel and of enormous capacity” (Baars, 1993).

## Gestalt, consciousness and general intelligence

Gestalt perception refers to how the human mind organises sensory input into meaningful wholes rather than perceiving individual components and their relationships. The term “Gestalt” originates from the early 20th German Gestalt Psychology movement, which insisted that gestalt, what we might today call holistic, aspects of an object, are primary to perception. Consider the two sentences “The pen is filled with ink” and “The sheep are in the pen.” The same word, “pen,” has completely different meanings instantly grasped in each sentence. Its context and gestalt properties define the word’s meaning. Optical illusions, such as the impossible or Penrose triangle, also illustrate the gestalt nature of perception. It appears to be a three-dimensional object that is physically impossible to construct in real life. The illusion only works because we see the object as a holistic whole, a gestalt, rather than merely an arrangement of lines and angles.

**Table 1:** 

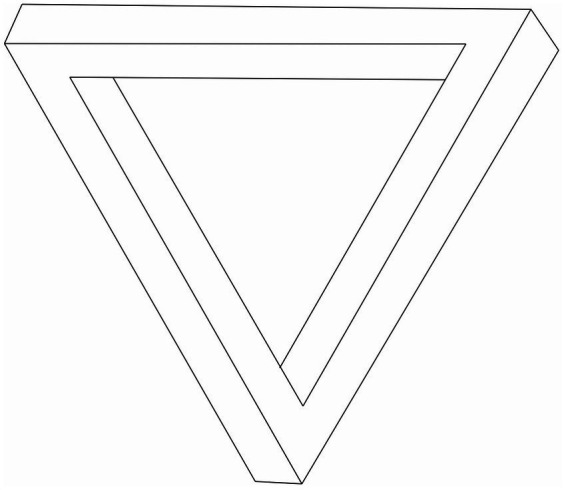

Conventional AIs and computers struggle to see what is wrong in objects such as the impossible triangle (Shahgir et al., 2024; Zhang and Yoshida, 2024; Maksymov, 2024), a problem likely related to why they cannot solve the rope caught in the wheel of a bicycle problem. A computer looking at an impossible triangle and a robot trying to untangle a rope from a bicycle wheel face a surprisingly similar situation: the challenge of reasoning about the physical world (Lake et al., 2017). Both require more than raw computation; they demand an understanding of geometry, physics, and cause-and-effect relationships that our conscious minds understand, but computers struggle with Leek et al. (2022) and Kuipers (2000).

When we see an impossible triangle, we instantly recognise something is wrong in our conscious mind because we see how it contradicts real-world geometry. Our brains, trained by a lifetime of interacting with physical objects, detect the paradox at the gestalt level of the whole object. A computer sees only a collection of lines and angles. A basic image recognition system, such as a convolutional neural network, might identify it as a “triangle-like shape” without understanding its fundamental impossibility. The contradiction would become clear only when an AI attempted to reconstruct it in three dimensions or simulate how its edges should connect. In contrast, in the gestalt sense, the human mind instantly knows that there is something fishy about the impossible triangle before we examine the connections between its edges and corners.

A similar issue arises when a robot might try to untangle a rope from a bicycle wheel. To a human, the solution is intuitive. We instinctively understand forces, tension, and the sequence of actions needed as a gestalt, whether, for example, to pull, loosen, or rotate the wheel first. We can even imagine different strategies before acting, simulating the operations as gestalts in our heads. But a computer lacks this kind of gestalt reasoning. Unlike humans, it does not possess an integrated internal model of how objects behave in the real world, a gestalt.

This kind of reasoning, gestalt reasoning, only operates in our conscious mind but is essential for general intelligence in the real world. We could never untangle a rope from a bicycle wheel nonconsciously, just as our nonconscious mind would never appreciate that the Penrose triangle is impossible or to understand poetry. So, what is this system that implements the general intelligence that we call our conscious mind?

## What is consciousness, and how does it work?

In his 1994 book, The Astonishing Hypothesis, the Nobel Laureate, Francis Crick, proposed a route towards a scientific understanding of consciousness by first identifying which features of the brain, its structures or dynamics, most often correlate with conscious thinking. In the succeeding decades, it was discovered that the synchronous firing of neurons is the most reliable *neural correlate of consciousness* or NCC. For example, several decades ago, work conducted by Wolf Singer and colleagues demonstrated that neurons in the monkey brain fire synchronously when the animal attends to the stimulus (Kreiter and Singer, 1996). Many subsequent studies have also shown that neural synchrony correlates with human conscious perception. For example, a subject’s neural synchrony patterns correlate with conscious recognition of, for example, the alternative perceptions of the face-vase illusion (Lutz et al., 2002; Bartels, 2009). Recent work has demonstrated that conscious auditory perception is correlated with long-range synchrony of gamma oscillations (Steinmann et al., 2014). Synchronisation between the anterior and posterior cortex has been shown to correlate with the consciousness levels of patients who have suffered traumatic brain injury (Leon-Carrion et al., 2012). But why should it make a difference to brain computation if neurons fire synchronously or asynchronously?

In papers published in 2002, McFadden (2002a,b) and Pockett (2002) independently proposed that consciousness correlates with synchronously firing neurons because the substrate of consciousness is not brain matter but the equally physical but immaterial brain’s EM field generated by synchronous neuron firing (McFadden, 2002a,b; Pockett, 2002). Since then, many related EM field theories of consciousness (EMF-TOCs) have been proposed (McFadden, 2002a; Pockett, 2002; Zhakenovich et al., 2016; Hales, 2017; Keppler, 2021; McFadden, 2020; Fingelkurts et al., 2013; Hunt and Schooler, 2019; John, 2002; Barrett, 2014; Pockett, 2012). The various EMF-TOCs share the claim that the substrate of consciousness is the brain’s EM field but differ in important details, so, whilst acknowledging the variety of EM-TOCs, in this paper, I will henceforth only refer to my own CEMI field theory.

The claim that the substrate of consciousness is the brain’s EM field may seem bizarre, but is it any more outlandish than the claim that the matter of the brain, essentially meat, is conscious? We can be sure that consciousness is a brain property, so it has to be instantiated in some physical substrate in the brain. Placing consciousness in the matter of the brain inevitably leads to the binding problem of understanding how information encoded in discrete units of matter in different parts of the brain is unified in the conscious mind. The binding problem evaporates once we accept that the substrate of consciousness is the brain’s EM fields, as EM fields are always unified. This is what we mean by a field. Physical fields integrate distributed information, making it available everywhere within the field. For example, the gravitational field at the soles of our feet, our weight, integrates our body’s mass with the entire planet’s mass. Similarly, the EM field at the membrane of a brain neuron integrates the information encoded by that particular neuron with all of the brain’s synchronously-firing neurons. The CEMI field theory proposes that the sum of these integrations across all synchronously-firing neurons in the human brain is the conscious mind.

## Evidence for EM-field based communication in the brain

There is no doubt that most of the brain’s information processing goes through its wires, the neuronal-synaptic route of action potentials that travel down the neuronal axon to initiate synaptic neurotransmitter release between neurons that trigger action potentials in downstream neurons. The endogenous EM fields generated by this neuronal firing are weak and would typically be unable to influence neural firing. Yet, as the philosopher Daniel Dennett noted, “consciousness is … a trickle of information that wins the competition for attention in a vast unconscious system” (Goldwyn and Rinzel, 2016). Potential changes of less than one millivolt across the neuronal membrane can modulate neuronal firing (Schmitt et al., 1976). Moreover, opening a single ion channel may be sufficient to trigger firing for neurons poised close to the firing potential (Arhem and Johansson, 1996). This degree of sensitivity suggests that tiny changes in membrane potential due to fluctuations in the brain’s endogenous EM field, also called local field potentials, may influence the firing of neurons that are already close to firing, potentially accounting for the “trickle” of conscious information processing that we experience in the human brain.

Evidence that neurons may communicate through EM field interactions, sometimes called ephaptic transmission, was scanty when EMF-ToCs were first proposed in 2002, but since then, both modelling (Ma et al., 2019; Tranchina and Nicholson, 1986; Abdeen and Stuchly, 1994; Goldwyn and Rinzel, 2016) and a wealth of experimental evidence (Frohlich and McCormick, 2010; Fujisawa et al., 2004; Anastassiou et al., 2011; Chiang et al., 2019; Anastassiou and Koch, 2015; Qiu et al., 2015; Han et al., 2018; Shibata et al., 2025; Martin and Kullmann, 2023; Van Horn et al., 2023) has accumulated, which demonstrates that the brain’s endogenous EM fields, local field potentials, play an essential role in communicating between brain neurons, even prompting some researchers to propose “that our visual experience may at least sometimes be coming through in waves” (Mathewson et al., 2011). Wave-like propagation of information in the brain is also consistent with recent brain mapping studies highlighting the importance of brain geometry, rather than just neuronal connectivity, in brain function (Van Horn et al., 2023). It “confirm[s] predictions that the close link between geometry and function is explained by a dominant role for wave-like activity” (Pang et al., 2023). In the brain a 2025 study in fruit flies discovered that an evolutionarily conserved cation neuronal membrane channel tunes the sensitivity of taste neurons to ephaptic (EM field) transmission (Lee et al., 2025). This ephaptic-sensitive channel, the HCN2 channel, could be genetically replaced by the human HCN2 channel. These findings suggest that ephaptic neural transmission in the brain is a very ancient neural communication mechanism conserved across animal species. In humans, the HCN2 and HCN1 channels are involved in pain reception (Dini et al., 2018) and are being actively investigated as drug targets to alleviate pain. Synchronised firing mediating EM field-based neuronal transmission has recently been proposed as both “necessary and sufficient for consciousness of pain” (Ambron, 2023a; Ambron, 2024; Ambron, 2023b).

Another potential role of the brain’s EM field is as the substrate of working memory, the cognitive system responsible for temporarily holding and manipulating information needed for complex mental tasks such as reasoning, learning, and decision-making. Its key features are its limited capacity, ability to handle only a few (around 4–7) items at a time, its short retention time of only seconds, and its capability to process its contents. These features are difficult to equate with a neural substrate, particularly when also considering representational drift (Rule et al., 2019). This is the phenomenon whereby the neural representation of an object in working memory, say an apple, changes over time, challenging the classical view that working memory relies on hard-wired neuronal connections. In their paper entitled “Beyond dimension reduction: stable electric fields emerge from and allow representational drift” Pinotsis and Miller (2022) and Pinotsis et al. (2023) describe studies which reveal that, although the neurons encoding a memory change from trial to trial, stability of working memory emerges at the level of the brain’s electric fields, as detected by EEG (Pinotsis and Miller, 2022). The authors conclude that the substrate for working memory is the brain’s EM field rather than its neurons. In their 2023 paper (Pinotsis et al., 2023), the authors go on to propose that “electric fields sculpt neural activity and ‘tune’ the brain’s infrastructure.” The higher level of correlation found in their studies between the contents of working memory and the brain’s EM fields, rather than the state of the brain’s matter-based neurons, represents a considerable challenge to neuronal accounts of working memory but is entirely consistent with the CEMI field theory.

Another key feature of the CEMI is its proposed role in driving conscious actions, forming the basis of our (free) “will.” This influence is illustrated in the adjacent figure in which three neuronal inputs, A, B, and C generate outputs, A, B and C. In the top panel, the neurons are firing asynchronously, causing destructive EMF interference and zero net field so the action delivered by the A, B, C outputs is non-conscious. In the bottom panel, the neurons are firing synchronously, causing constructive interference and a net EM field, the CEMI field, that integrates the A, B, C inputs to generate an EMF-influenced-output (Babcock and Kattnig, 2021) that is experienced as a willed action.

**Table 2:** 

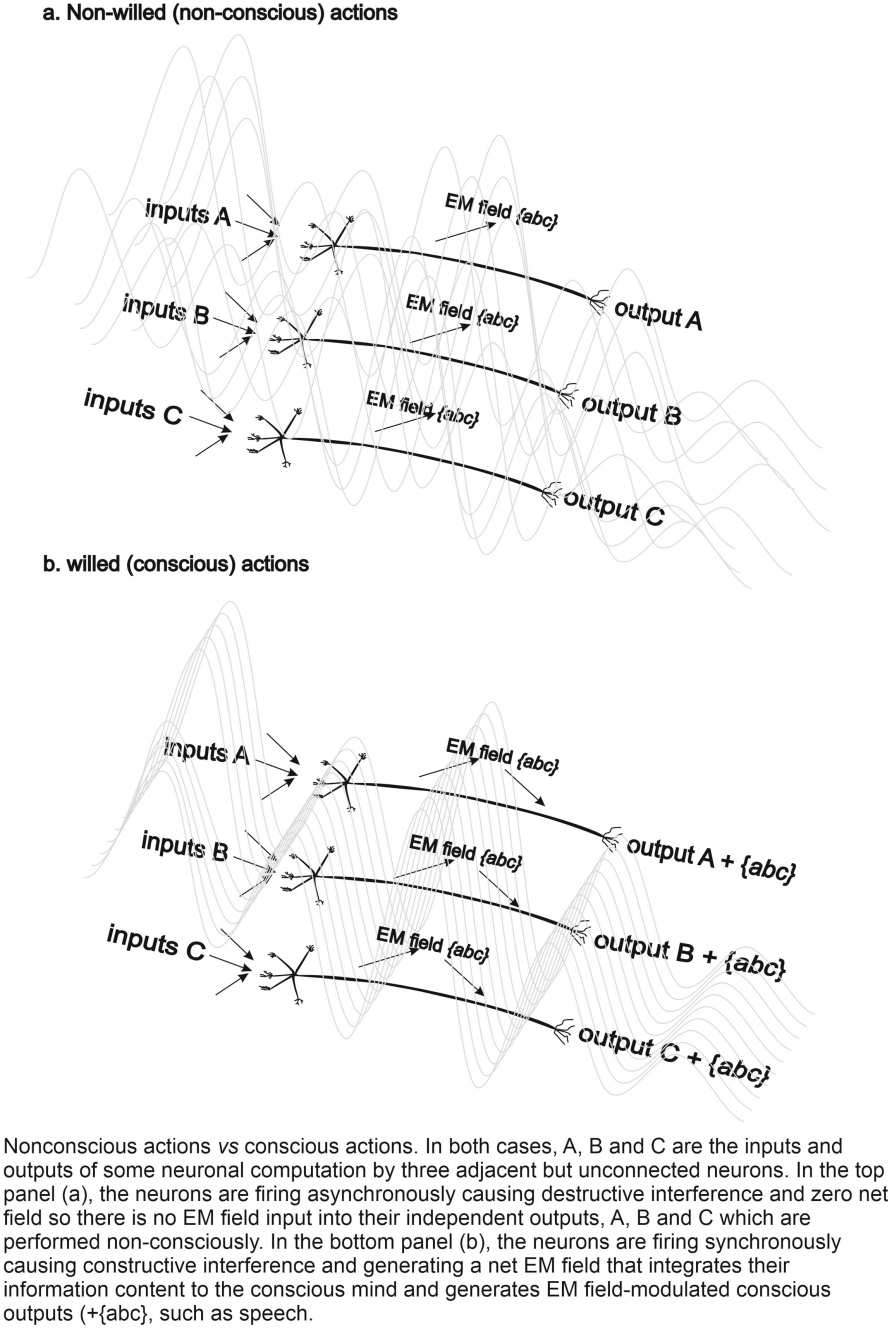

A striking parallel to this idea is in brain-computer interfaces (BCIs) or brain-machine interfaces (BMIs), which help paralysed individuals control prosthetic limbs or speech synthesisers. These systems often use EEG signals—the external expression of the brain’s EM field—as the interface between brain activity and machine output (Mahmood et al., 2021), effectively implementing the same informational loop from EM field-encoded neuronal information to motor actions as proposed by the CEMI field theory. Further into the future are efforts, such as those by Elon Musk’s company Neuralink (Musk, 2019), to enhance human capabilities using neural implant electrodes inserted into the brain to record the brain’s extracellular EM field. Though still limited, the success of BMIs (Kawala-Sterniuk et al., 2021) shows that EM field-encoded information can direct willed actions. The CEMI field theory suggests that, in healthy individuals, the brain’s endogenous EM field similarly directs the body’s conscious actions. It also predicts that users of EEG-controlled CMIs and BMIs will feel their actions are consciously willed. Users of the first commercial CMI,^3^ which uses EEG sensors above the visual cortex to detect focus and control digital environments, report the system feels “natural,” as if they will the actions themselves.

In summary, there is now abundant evidence that neurons not only communicate via the brain’s wires via the action potential-synaptic transmission route but also via endogenously generated brain EM fields: the brain’s wireless mode. But how might EM field information be processed in the brain?

## EM field integrated information processing in the brain

The CEMI field theory proposes that when groups of neurons fire in sync, their signals combine through constructive interference, amplifying and integrating their information and effectively uploading it into the brain’s endogenous electromagnetic EM field: the brain’s *input field*. Note that although several neurons may be firing in sync, they may nevertheless encode different information due to their position in the brains. A neuron excited by the colour red in the visual cortex may fire at the same frequency as a neuron in the auditory cortex that is stimulated by the middle C musical note, but, as highlighted above, geometry plays a vital role in the brain, one that is instantiated in the geometry of the brain’s EM field, itself generated by the firing of disparately-located synchronously-firing neurons. The shape and dynamic structure of the brain’s endogenous EM field is, of course, what is routinely detected and measured by medical diagnostic technologies such as EEG (electroencephalography) and MEG (magnetoencephalography), both of which are commonly used to monitor states of consciousness (Voss and Sleigh, 2007; Musialowicz and Lahtinen, 2014).

In the brain’s endogenous EM field, input signals will be processed through constructive and destructive interference both with each other and with the brain’s existing EM field state to generate a new EM field that integrates and processes the inputs in the context of the preexisting field (McFadden, 2020). The processing essentially performs an analogue computation on the incoming neural firing data, sometimes referred to as “field computing” or “quantum-like computing (MacLennan, 1999; MacLennan, 2022) to generate an *output field*. Note that this quantum-like computing, unlike quantum computing consciousness paradigms (Hameroff and Penrose, 1996; Hameroff, 2001; Hagan et al., 2002; Hameroff et al., 2002; Fisher, 2015; Swift et al., 2018) does not require exotic states of matter, just the interaction of classical EM fields.

CEMI field theory further proposes that brain neurons *upload* the processed output field through their voltage-gated ion channels, such as the human HCN2 channel mentioned above, which modulates their firing rates. HCN2 channels are widely distributed in the human brain, found in the cortex, hypothalamus and sensory neurons and are crucial in regulating neuronal excitability and rhythmic activity. They also play a pivotal role in pain perception, particularly neuropathic and inflammatory pain. This modulation influences neural activity and enables reports of the contents of the conscious mind via motor functions such as speech.

It is possible that the brain’s endogenous EM field could also contribute to back-propagation as its influence on neural firing rates will provide feedback on neural signalling that would be non-local, field-based, and potentially capable of modulating firing probabilities of multiple neurons simultaneously. It would lack the algorithmic precision and differentiable structure required for strict backpropagation as implemented in machine learning and various neural network architectures, but it could contribute to adaptive learning by reinforcing neural assemblies that produce coherent EM field patterns (that represent conscious thoughts) and by modulating synaptic plasticity in circuits exposed to reinforcing EM patterns.

By integrating distributed neuronal information in a physical field, EM-TOCs provide a solution to both the binding problem and the problem of understanding why our conscious mind is a serial computer: EM fields are always singular, so new information entering the brain’s EM field will always interfere with existing information already encoded in that field. The loop of brain-based neuronal information being first transmitted into the brain’s EM field and, after field-based information processing, being reflected back into brain neurons is also self-reflexive and thereby constitutes what the cognitive scientist Douglas Hofstadter called a Gödelian “strange loop” (Hofstadter, 1979; Hofstadter, 2007) and proposed to be central to conscious cognition. But how might EM field computing be implemented in an AI?

## Potential routes towards EM field integrated information processing in an AI

It is not surprising that modern computers are not conscious and are thereby (according to the CEMI field theory) incapable of general intelligence. Electronic engineers go to great pains to avoid EM field communication between the electrical components of computers as intra-system electromagnetic interference (EMI) and radio-frequency interference (RFI) may drastically affect the *in situ* performance of advanced integrated circuits (ICs) and electronics more generally (Slattery and Skinner, 2011). However, evolution did not have the benefit of electronic engineers, so, as biological brains grew bigger and became more densely packed with neurons, EM field interference between neurons became inevitable. Where EM field interference impaired function and thereby reduced fitness, natural selection would have acted to minimise EM field interference to develop EM field-insensitive neural circuits. Where EM field interference enhanced function and thereby increased fitness, natural selection would have acted to optimise EM field interference to develop EM field-sensitive neural circuits. The CEMI field theory proposes that these two systems became the nonconscious and conscious minds, respectively. In the following section, I provide some speculative paths towards generating a conscious AI capable of general intelligence.

A possible route towards building an EM field-sensitive AI would be to emulate the evolution of the human brain by employing evolvable hardware approaches (Trefzer and Tyrrell, 2015) that could capture EM field-sensitive circuitry. The School of Cognitive & Computing Sciences (COGS) group at the University of Sussex may have inadvertently accomplished this (Davidson, 1997; Thompson, 1996). The team artificially evolved a field-programmable gate array (FPGA). Starting from a population of randomly configured chips, they selected configurations that performed slightly better at solving the toy task of distinguishing between two musical tones. After 5,000 generations, they had artificially evolved a chip that could efficiently accomplish this task. However, when they examined its circuit diagram, they discovered that some of its components, which, if removed, impaired function, were not connected by wires to either inputs or outputs. The team concluded that their evolutionary process had not only optimised the wired connection of the chip but also likely harnessed EMF coupling between components of the FPGA chip. Similar approaches that took advantage of more advanced chips, coupled with advanced modelling studies, could, at the very least, provide new insights on how EM field interactions could be incorporated into digital computing devices.

The human brain took around 500 million years to evolve from simple animal nervous systems. Modern electronics could likely move faster, but it would be unlikely to deliver the computational capacity of the human brain in any reasonable timeframe. More likely, EMF coupling, perhaps informed by insights gained from evolutionary approaches, could be implemented into the architecture of a conventional computer to build a hybrid digital-EMF, HyDEMF AI.

## Possible design of HyDEMF computing architecture

A very sketchy architecture would combine:

1. A Boolean logic processing layer that generates and is modulated by endogenous EM fields.

The AI’s fundamental processing unit could operate a traditional computing Boolean logic layer, analogous to neuronal/synaptic processing in the human brain. This layer might include:

- Digital logic gates that process symbolic information deterministically.
- Neural network architectures that handle pattern recognition and statistical inference.
- Sequential processing mechanisms, ensuring structured, rule-based computation.
- Generation of endogenous EM fields, which contribute to the EM field layer.
- Units influenced by the EM field interaction layer, modifying logic gate activity through EM interference effects.

Hardware components for this layer could include:

○ FPGA and ASICs (Application-Specific Integrated Circuits) optimised for hybrid computing.
○ Neural Processing Units (NPUs) capable of executing both conventional logic operations and generating structured oscillatory signals.
○ Integrated circuits with electromagnetic field sensors to allow for dynamic field-driven computation. This layer will execute traditional AI algorithms, performing structured learning tasks and handling discrete symbolic representations whilst integrating field-based modulation.

**Table 3:** 

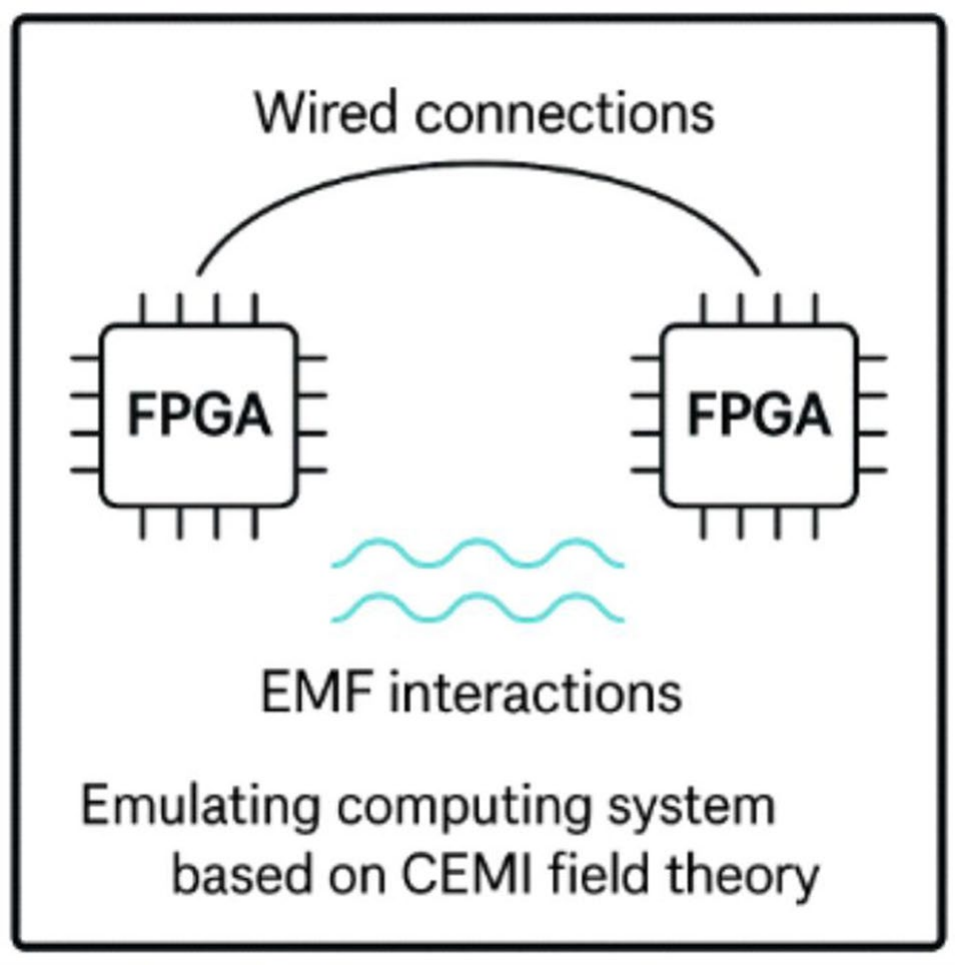

2. An EM field layer that processes information through wave-based interference.

Unlike the Boolean logic layer, this system will operate through continuous field dynamics rather than discrete logical states. Key features include:

- Electromagnetic field generation from synchronised computational elements.
- Constructive and destructive interference patterns enabling wave-based computation.
- A continuous dynamical representation of information, independent of discrete processing.

The EM field layer is a global computational workspace, dynamically processing information via EM field interactions. Hardware components could include:

○ Dielectric resonators and metamaterial-based processors that enable precise EM field control.
○ Reconfigurable electromagnetic waveguides capable of directing and modulating field interactions dynamically.
○ Embedded antennas within logic circuits to facilitate EM wave interference computations.

3. An EM field interaction layer that modulates the Boolean logic layer based on feedback from the EM field layer.

This layer plays a modulatory role, influencing the Boolean logic layer based on the states of the EM field layer. Key characteristics include:

- Field-based feedback loops that regulate Boolean logic operations.
- Non-local information processing, where global field effects influence local computations.
- Wave-mediated modifications of logic gate operations that introduce additional computational influence beyond Boolean logic.

This layer allows for real-time adaptation of the Boolean processing layer based on the EM field environment by modulating logic gate behaviour. This feedback loop mirrors the proposed role of the brain’s EM field by integrating and influencing neural activity, potentially leading to emergent, self-organising intelligence in AI systems. Possible hardware components include:

○ EM-sensitive transistors or spintronic components that allow logic gates to be modulated by the surrounding field.
○ Hybrid optical-electromagnetic computing elements that bridge Boolean logic with wave-based processing.
○ Quantum-dot or nanoscale EM-field detectors for fine-grained sensing and adaptation of logic states.

This three-layer model could allow for emergent computational behaviours and an alternative paradigm for AI cognition. A system incorporating these hardware components would enable real-time interaction between digital logic and wave-based EM field computation, paving the way for emergent AI cognition. Modelling the electrodynamics of designed circuits (Taflove, 1995; Sagar et al., 2021) and comparison with electrodynamic modelling of live neurons (Muni et al., 2022) would complement engineering approaches.

## Possible advantages of hybrid digital-EMF, HyDEMF, computing architecture

An AI system based on these principles would require a fundamental shift from the discrete, modular architecture of conventional computing towards a system that integrates continuous, field-based interactions. In a hypothetical hybrid digital-EMF AI, information processing would not rely solely on static logic gates but would involve dynamic patterns of constructive and destructive interference within an electromagnetic substrate. These interference patterns could encode complex relationships between data points in a way that more closely resembles human cognition. For example, in traditional AI, an image recognition algorithm processes pixels individually, extracting features through layers of a neural network to determine what an object is. In an EM field-based AI, the entire field might encode holistic representations of the entire image, much like how the brain perceives an object as a unified whole, a gestalt, rather than a collection of independent pixels.

One potential advantage of such a system is its ability to integrate real-time information without requiring sequential, step-by-step processing. Conventional computers process instructions in a linear or parallel fashion, with each operation following a predefined logic path. An EM field-based AI could compute through wave interactions, where multiple pieces of information influence each other simultaneously, similar to holographic information processing (Frauel et al., 2006). This could allow for a more fluid and adaptable form of cognition, particularly useful in tasks requiring rapid situational awareness, such as autonomous decision-making or natural language understanding.

Feedback modulation from the EM field interaction layer could also introduce adaptive learning mechanisms beyond traditional weight updates in artificial neural networks. This could enable more robust learning in uncertain environments, flexible cognitive responses without retraining and the potential emergence of self-organising intelligence.

The HyDEMF system may also deliver improved energy efficiency. The brain operates at approximately 20 W (watts) of power, roughly the same as a small lightbulb. In contrast, modern supercomputers and AI models, such as GPT-like networks, consume far more power. For instance, the Fugaku supercomputer in Japan consumes around 28 MW of power. Even a high-end graphics processing unit cluster for AI training can consume hundreds of kilowatts. So the human brain is about a million times more power-efficient than modern AI systems performing similar cognitive tasks.

Although the source of the brain’s impressive energy efficiency is not known, it is plausible that EM field computation provides at least some of its low-energy computational capacity. For example, using fields to transmit information allows for less reliance on extensive physical wiring, which in brains is a major volume and energy cost. Field-based integration could allow for more compact, modular architectures that could reduce overall signal travel and metabolic demand. Moreover, although the EM field might only rarely directly cause neurons to fire, it may instead bias neurons towards or away from firing depending on their membrane potential proximity to threshold. A small EM field influence, if widespread, could coordinate or modulate ensembles without generating spikes, which are energetically expensive.

Introducing wave-based field computation may enable AI efficiencies closer to biological brains.

Of course, many challenges will likely be associated with implementing an AI system based on HyDEMF architecture. For example, electromagnetic waves can encode information but are also susceptible to environmental interference. Unlike transistors, which operate with well-defined electrical states, EM fields are inherently fluid and can be influenced by external signals. Ensuring that an AI system based on EM fields can maintain reliable processing without being disrupted by background noise may require EMF shielding together with sophisticated error correction mechanisms, possibly inspired by the brain’s own ability to filter relevant signals from irrelevant ones in a noisy, fluid environment.

Developing field-sensitive logic gates or hybridised FPGA-like architectures capable of EM interaction modulation to support field-based computation would also be a challenge, as would establishing a suitable learning framework. The absence of established learning mechanisms for field-based computation makes it unclear how such a system would autonomously refine its processing capabilities. Could a system akin to backpropagation emerge naturally in an EM-mediated AI? Would an HyDEMF AI need to have a body to develop human-like reasoning? These are open questions.

## Conclusion

The CEMI field theory, and related EMF theories of consciousness (TOCs), differ from nearly all other established TOCs by proposing that the physical substrate of consciousness is the brain’s EM field rather than its matter. The theory does not deny that much, probably most, of what the brain does goes through its matter-based *wires* of dendrites, neuronal axons and synapsis but proposes that its “stream of consciousness” (James, 1892), which is likely more of a trickle compared to nonconscious neural processing, flows in the brain’s electromagnetic field. The theory solves the binding problem, explains why our mind operates in two very different modes and why current AIs are nonconscious. In previous publications, I have compared CEMI field theory with other TOCs, most recently the two current leading TOCs, integrated information theory and global workspace theory (McFadden, 2023a), arguing that CEMI field theory outperforms both. Although it may seem strange to propose that consciousness is an invisible field, is it any stranger than proposing that consciousness is, essentially, meat, the matter of the brain (McFadden, 2023b)? Matter and fields are both physical entities. Indeed, in quantum theory, all matter particles are, ultimately, oscillations of fields. The CEMI field theory is a physicalist, though not materialist, TOC.

The Nobel Prize-winning engineer and physicist, Richard Feynman, famously insisted that “what I cannot make I do not understand.” On Feynman’s criterion, we do not understand consciousness as we have not created it any artificial device including neural networks with informational processing architecture that mirrors the wiring of the human brain and on computers with computational power and speed that are now competitive the human brain. Also applying Feynman’s criterion, the only definitive proof of a particular TOC will be the creation of a synthetic consciousness based on the principles of that theory. In this paper, I take the first steps in that direction on behalf of the CEMI theory by proposing a novel computational architecture that the theory predicts will be conscious.

By building a system that integrates wired logical structure with field-based adaptability, a HyDEMF AI architecture could bridge the gap between conventional AI and human cognition, potentially unlocking new levels of intelligence beyond classical computing paradigms, including general intelligence and creativity. There are, of course, significant technical challenges to implementing such a system. Biological brains have evolved over millions of years alongside endogenous EM fields, with neural structures optimising how signals propagate and interfere. Replicating this in artificial hardware could require new materials science, physics, engineering and computational theory breakthroughs.

Despite these challenges, research into alternative computing models continues to push the boundaries of what is possible in AI and cognitive science. Some experimental work has explored optical computing, where light waves, rather than electrical currents, are used for information processing (McMahon, 2023). EM field-based computing also shares some similarities with holographic information processing, such as using interference patterns to manipulate data (Frauel et al., 2006). It is possible that AI systems could incorporate novel information-processing substrates to achieve more human-like intelligence.

In addition to practical applications in AI, the implications of Gestalt-based field computation could clarify our understanding of consciousness. The CEMI field theory proposes that consciousness is what EM field-based computing *feels like* from inside the EM field. We are aware of the stream of information flowing through the brain’s EM field because we *are* that stream of information flowing through the brain’s EM field. It is only there, in the ethereal material that we call electromagnetic fields, where brain information is unified to form complex thoughts that can understand poetry or work out how to fix a bicycle with a rope tangled in its wheels and generate motor outputs that can communicate those thoughts in speech, poetry painting, music, dance or other creative outputs of the human mind.

If the CEMI theory is correct, and the brain’s EM field plays a fundamental role in generating conscious experience, then developing AI systems replicating this mechanism might bring us closer to creating machines with genuine awareness. Could such a system feel happiness, sadness or pain? Would such an AI possess subjective experiences or merely simulate them? How would we measure or verify the presence of consciousness in an artificial system? These are profound philosophical and ethical questions that, it seems likely, could only be answered by a conscious AI.

## Data Availability

The original contributions presented in the study are included in the article/supplementary material, further inquiries can be directed to the corresponding author.

## Footnotes

^1^I leave open the question of non-human intelligence/consciousness.

^2^I refer to actions as “nonconscious” if we perform them without awareness. They range from the contractions of muscles whilst we walk or cycle, blinking, constructing grammatically correct sentences or controlling the muscles of out lips, tongue and larynx to utter these sentences. The meaning of the term has little of no relation to the term “unconscious” as used in psychoanalysis.

^3^ https://www.next-mind.com/
